# Supplementary material for: The Functional Association of ACQOS/VICTR with Salt Stress Resistance in Arabidopsis thaliana Was Confirmed by CRISPR-Mediated Mutagenesis
Source: Int J Mol Sci. 2021 Oct 21;22(21):11389. doi: 10.3390/ijms222111389 (PMC8583979; doi:10.3390/ijms222111389)
Supplement: Supplementary file 1 [file ijms-22-11389-s001.zip › Figure S1.pdf]

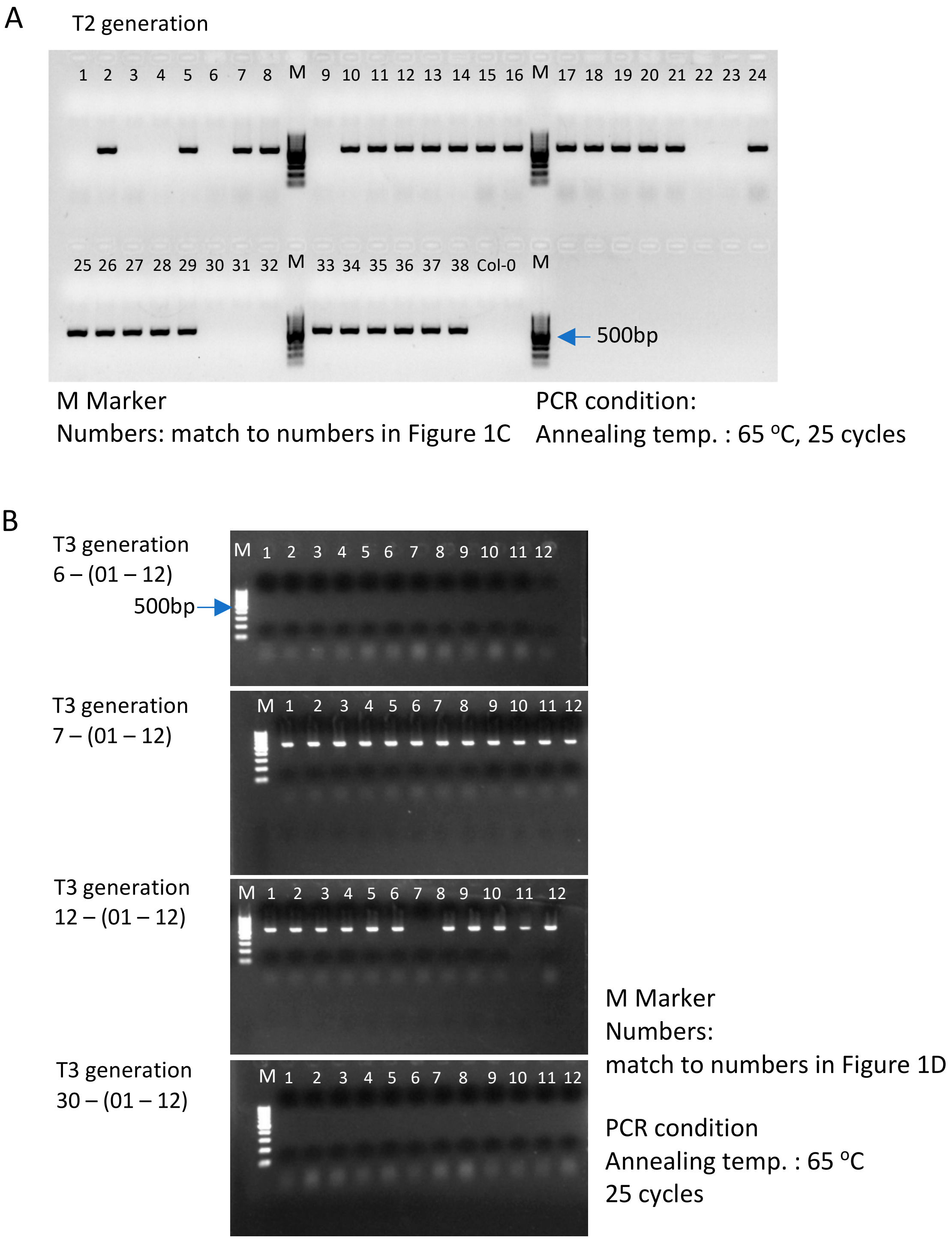

Figure S1. Electrophoresis of PCR product amplified with primers for T-DNA detection in T2 and T3 generation plants. We amplified the herbicide resistance gene (552 bp) in the vector. The primer sequences are in Table S1.
