# Supplementary material for: The Functional Association of ACQOS/VICTR with Salt Stress Resistance in Arabidopsis thaliana Was Confirmed by CRISPR-Mediated Mutagenesis
Source: Int J Mol Sci. 2021 Oct 21;22(21):11389. doi: 10.3390/ijms222111389 (PMC8583979; doi:10.3390/ijms222111389)
Supplement: Supplementary file 1 [file ijms-22-11389-s001.zip › Table S1.pdf]

Table S1. Primer list used in this study

T-DNA detection PCR

Primer name	SEQ(5'-3')
Basta_FP	TCAGATTTCGGTGACGGGCA
Basta_RP	ATGAGCCCAGAACGACGC
Cas9_center_F	GAAGATCGAGAAGATCCTGA
Cas9_center_R	GAAGTCCTTGTCCTTGATGA

Amplicon deep sequencing

Primer name	SEQ(5'-3')
SG75_F for G1 nested PCR	TTGTTTGGGTAAATAATCATCGTTT
SG76_R for G1 nested PCR	CACCCAGTACTCCTTTTTTTCTT
SG77_F for G2 nested PCR	CATTATTAGATACATCTTGAAATAAAATTG
SG78_R for G2 nested PCR	CACCCAGTACTCCTTTTTTTCTC
SG139 for G1 2nd PCR	ACACTCTTTCCCTACACGACGCTCTTCCGATCTGGTATATGGGGTTCCTCGGG
SG140 for G1 2nd PCR	GTGACTGGAGTTCAGACGTGTGCTCTTCCGATCTTCTTTTTTCCCCAAAATTTCCG
SG141 for G2 2nd PCR	ACACTCTTTCCCTACACGACGCTCTTCCGATCTTTAGCAAATTCATAGACAGAGC
SG142 for G2 2nd PCR	GTGACTGGAGTTCAGACGTGTGCTCTTCCGATCTTGATGAGAGTTTTCTGGTGC
