# Supplementary material for: The Functional Association of ACQOS/VICTR with Salt Stress Resistance in Arabidopsis thaliana Was Confirmed by CRISPR-Mediated Mutagenesis
Source: Int J Mol Sci. 2021 Oct 21;22(21):11389. doi: 10.3390/ijms222111389 (PMC8583979; doi:10.3390/ijms222111389)
Supplement: Supplementary file 1 [file ijms-22-11389-s001.zip › Table S2.pdf]

Table S2. Mutation efficiencies by amplicon deep sequencing.

T1 generation

Sample	AT5G46510(G1)			Sample	AT5G46520(G2)		
	Total read	Mutation	Indel ratio (%)		Toltal read	Mutation	Indel ratio (%)
Col-0	21793	0	0	Col-0	25657	2	0
S-1	20912	546	2.6	S-1	22218	649	2.9
S-2	18000	202	1.1	S-2	23448	2771	11.8
S-3	18747	120	0.6	S-3	24921	261	1
S-4	26946	689	2.6	S-4	21651	664	3.1
S-5	15133	279	1.8	S-5	22213	577	2.6
S-6	22235	207	0.9	S-6	19640	288	1.5
S-7	21894	596	2.7	S-7	19290	549	2.8
S-8	22717	576	2.5	S-8	33372	932	2.8
S-9	25653	160	0.6	S-9	34756	262	0.8
S-10	30200	30030	99.4	S-10	27419	27287	99.5
S-11	30202	533	1.8	S-11	27742	655	2.4
S-12	25415	1216	4.8	S-12	34671	2062	5.9

T2 generation

Sample	AT5G46510(G1)			Sample	AT5G46520(G2)			T-DNA detection
	Total read	Mutation	Indel ratio (%)		Toltal read	Mutation	Indel ratio (%)	
1	11718	32	0.27	1	7781	0	0.00	-
2	13162	11411	86.70	2	15907	12616	79.31	+
3	13314	79	0.59	3	4841	2266	46.81	-
4	16019	34	0.21	4	11329	13	0.11	-
5	16289	16134	99.05	5	14310	14295	99.90	+
6	19368	77	0.40	6	16638	7570	45.50	-
7	15000	14953	99.69	7	14099	14082	99.88	+
8	11777	9455	80.28	8	8507	3279	38.54	+
9	16934	9	0.05	9	13528	7	0.05	-
10	16607	16333	98.35	10	11139	11071	99.39	+
11	17445	17426	99.89	11	14091	14080	99.92	+
12	16189	16175	99.91	12	10515	10506	99.91	+
13	14413	14386	99.81	13	17624	17451	99.02	+
14	14095	13857	98.31	14	13332	13319	99.90	+
15	16188	15558	96.11	15	14958	14548	97.26	+
16	12315	12310	99.96	16	12034	12012	99.82	+
17	8917	8909	99.91	17	13033	12907	99.03	+
18	13500	12948	95.91	18	15299	15062	98.45	+
19	11376	7683	67.54	19	11737	9406	80.14	+
20	11759	9513	80.90	20	12808	8131	63.48	+
21	10010	10002	99.92	21	14444	14435	99.94	+

22	15025	26	0.17	22	16344	7	0.04	-
23	10290	5	0.05	23	14131	5	0.04	-
24	6323	6273	99.21	24	12057	11973	99.30	+
25	11021	11017	99.96	25	16199	16109	99.44	+
26	7910	7910	100.00	26	16779	16710	99.59	+
27	17122	17090	99.81	27	16170	16044	99.22	+
28	16177	14028	86.72	28	14095	12868	91.29	+
29	15344	15325	99.88	29	15822	15808	99.91	+
30	14463	9202	63.62	30	18400	372	2.02	-
31	16461	10	0.06	31	10991	116	1.06	-
32	12163	7	0.06	32	10370	73	0.70	-
33	15022	14451	96.20	33	12755	11316	88.72	+
34	9210	9203	99.92	34	8942	8940	99.98	+
35	13673	13542	99.04	35	8533	8529	99.95	+
36	11434	11421	99.89	36	15606	15581	99.84	+
37	13865	13856	99.94	37	10474	10446	99.73	+
38	15465	15367	99.37	38	13230	13120	99.17	+

T3 generation

Sample	AT5G46510(G1)			Sample	AT5G46520(G2)			T-DNA detection	in/del in G1	in/del in G2
	Total read	Mutation	Indel ratio (%)		Toltal read	Mutation	Indel ratio (%)			
6-01	2270	0	0	6-01	6413	6403	99.8	-		ins. A
6-02	2756	3	0.1	6-02	6632	6	0.1	-		
6-03	2284	2	0.1	6-03	6721	6719	100	-		ins. A
6-04	1984	0	0	6-04	5729	2974	51.9	-		
6-05	2798	4	0.1	6-05	5270	5263	99.9	-		ins. A
6-06	2534	2	0.1	6-06	5406	2610	48.3	-		
6-07	2536	0	0	6-07	4670	2324	49.8	-		
6-08	2130	3	0.1	6-08	5250	2676	51	-		
6-09	2738	0	0	6-09	5553	10	0.2	-		
6-10	3217	0	0	6-10	5633	10	0.2	-		
6-11	2532	0	0	6-11	5451	10	0.2	-		
6-12	2444	0	0	6-12	5101	5091	99.8	-		ins. A
7-01	2427	2427	100	7-01	5867	5865	100	+	ins. T	ins. T
7-02	2097	2097	100	7-02	6043	6028	99.8	+		
7-03	2389	2389	100	7-03	5838	5835	99.9	+	ins. T	ins. A
7-04	1963	1963	100	7-04	5714	5706	99.9	+	ins. A	ins. A
7-05	2271	2271	100	7-05	6502	6502	100	+	ins. T	ins. T
7-06	2769	2767	99.9	7-06	1736	1734	99.9	+	ins. T	
7-07	2208	2208	100	7-07	6008	5998	99.8	+	ins. T	ins. T
7-08	2095	2095	100	7-08	5413	4260	78.7	+	ins. T	
7-09	2221	2221	100	7-09	5904	5447	92.3	+	ins. T	
7-10	2160	2160	100	7-10	6361	6359	100	+	ins. T	
7-11	1999	1999	100	7-11	5872	5872	100	+	ins. T	ins. A

7-12	1782	1782	100	7-12	6095	6087	99.9	+	ins. T	ins. A
12-01	2242	2242	100	12-01	6948	6944	99.9	+	ins. A	ins. A
12-02	2635	2635	100	12-02	6399	6395	99.9	+		
12-03	2142	2142	100	12-03	6989	6987	100	+	ins. A	ins. A
12-04	1961	1961	100	12-04	5794	5791	99.9	+	ins. T	
12-05	2067	2067	100	12-05	5667	5667	100	+		
12-06	1994	1994	100	12-06	5943	5941	100	+		
12-07	2153	2153	100	12-07	5511	5509	100	-	ins. T	del. C
12-08	1760	1760	100	12-08	5878	5874	99.9	+		
12-09	2175	2175	100	12-09	5956	5954	100	+		del. C
12-10	2792	2792	100	12-10	2812	2812	100	+	ins. G	
12-11	2058	2058	100	12-11	1154	1150	99.7	+	ins. A	ins. A
12-12	1788	1785	99.8	12-12	3527	3514	99.6	+		
30-01	2807	1372	48.9	30-01	5416	2	0	-		
30-02	2527	2527	100	30-02	5932	2	0	-	ins. A	
30-03	2628	2626	99.9	30-03	5461	0	0	-	ins. A	
30-04	2198	1175	53.5	30-04	5531	0	0	+		
30-05	2869	2869	100	30-05	5617	0	0	-	ins. A	
30-06	3241	1724	53.2	30-06	6129	0	0	-		
30-07	2522	1334	52.9	30-07	5438	0	0	-		
30-08	2186	1170	53.5	30-08	5411	0	0	-		
30-09	1916	1914	99.9	30-09	4681	0	0	-	ins. A	
30-10	1627	0	0	30-10	4830	0	0	-		
30-11	1739	1739	100	30-11	4705	0	0	-	ins. A	
30-12	1476	1476	100	30-12	5142	0	0	-		
Col-0	1905	2	0.1	Col-0	4874	2	0	-		
